# Profiling the secretome: maternal obesity impacts redox and adipogenic signaling during neonatal mesenchymal stem cell adipogenesis

**DOI:** 10.1093/stmcls/sxag001

**Published:** 2026-01-10

**Authors:** Sofía Bellalta, Erika Pinheiro-Machado, Paola Casanello, Marijke Faas, Torsten Plösch

**Affiliations:** Department of Pathology and Medical Biology, University of Groningen, University Medical Center Groningen, 9713 GZ Groningen, The Netherlands; Department of Obstetrics, School of Medicine, Pontificia Universidad Católica de Chile, 8330024 Santiago, Chile; Department of Pathology and Medical Biology, University of Groningen, University Medical Center Groningen, 9713 GZ Groningen, The Netherlands; Department of Obstetrics, School of Medicine, Pontificia Universidad Católica de Chile, 8330024 Santiago, Chile; Department of Neonatology, School of Medicine, Pontificia Universidad Católica de Chile, 8330024 Santiago, Chile; Department of Pathology and Medical Biology, University of Groningen, University Medical Center Groningen, 9713 GZ Groningen, The Netherlands; Department of Obstetrics and Gynaecology, University of Groningen, University Medical Center Groningen, 9713 GZ Groningen, The Netherlands; Department of Obstetrics and Gynaecology, University of Groningen, University Medical Center Groningen, 9713 GZ Groningen, The Netherlands; Perinatal Neurobiology Research Group, School of Medicine and Health Sciences, Carl von Ossietzky Universität Oldenburg, 26129 Oldenburg, Germany

**Keywords:** maternal obesity, stem cells, fetal programming, adipogenesis, secretome

## Abstract

Recent studies evidence an altered bioenergetic profile and higher adipogenic commitment in the mesenchymal stem cells (MSC) from neonates of mothers with obesity. We hypothesize that these alterations may also affect the secretome of these cells. The aim of this study was to characterize the secretome of MSCs from the offspring of women with obesity compared to the ones from normal-weight women, both before and during adipogenesis. Wharton’s jelly-derived MSCs were isolated from newborns of normal-weight women (NW-MSC; Body Mass Index 18.5-24.5 kg/m^2^) and women with obesity (OB-MSC; Body Mass Index > 30 kg/m^2^) and cultured for 0, 5, and 21 days of adipogenesis. The secretome from these cells was collected during the three timepoints and characterized by mass spectrometry. Our findings reveal fundamental differences in the secretome profiles, primarily associated with pathways involved in cellular and metabolic processes. Maternal obesity was found to decrease redox capacity at day 0 but subsequently triggered a compensatory increase in redox proteins during adipogenesis of OB-MSCs. Additionally, OB-MSCs secreted higher levels of lipid synthesis-related proteins and proinflammatory adipokines, which may contribute to the dysregulated adipogenesis observed in obesity. These preliminary data indicate that maternal obesity programs the secretome of neonatal MSCs, supporting the hypothesis that maternal obesity imprints early progenitor cells and potentially dictates the future metabolic status of the offspring’s adipocytes.

## Graphical abstract

**Graphical Abstract:**
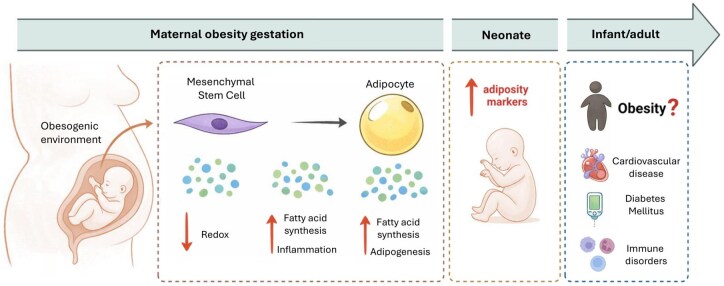

## Introduction

Significance statement Maternal obesity increases the risk of childhood obesity, yet its specific effect on early progenitor cells from the adipose tissue remains poorly understood. This study reveals that mesenchymal stem cells from neonates of mothers with obesity exhibit a distinct secretome profile, particularly in redox balance, lipid metabolism, and proinflammatory signaling, compared to the ones from normal weight mothers. Our preliminary findings suggest that maternal obesity programs the neonatal mesenchymal stem cells secretome, potentially predisposing offspring to adipose tissue dysfunction and metabolic dysregulation later in life. These provide important insights into the developmental origins of obesity and future metabolic health risk.

Mesenchymal stem cells (MSCs) hold a significant relevance within their cellular microenvironment.^1^ Central to their significance is their paracrine activity, which orchestrates communication with the cellular milieu to maintain homeostasis.^2^^,^^3^ Referred to as the MSCs secretome, this paracrine profile consists of soluble factors such as growth factors, hormones, cytokines, and metabolites, in addition to the release of extracellular vesicles.^3^ These vesicles transport an array of bioactive molecules, surface receptors, and signaling molecules, engaging with other cells.^4–8^ Thus, the secretome provides a novel approach to exploring the functional role of MSCs.

MSC function becomes particularly relevant in the context of maternal obesity, a condition strongly associated with an increased risk of childhood obesity in the offspring.^9^^,^^10^ While the mechanisms underlying the programming of fetal adipose tissue remain elusive. MSCs, as progenitor cells established during early embryonic development, are notably present in fetal tissues like the umbilical cord.^11–13^ Current findings indicate that neonatal MSCs, the embryonic precursor cells for adipocytes, exhibit early signatures of an adipogenic commitment when influenced by maternal obesity.^14–16^ MSCs from the umbilical cord serve as an efficient model for studying the underlying mechanisms driving adipogenic commitment of MSCs in obese pregnancies.

Adipogenic commitment from MSCs is accompanied by significant metabolic reprogramming, including increased mitochondrial activation and mass.^17–19^ During early adipogenic commitment (day 0-5), this mitochondrial activation triggers an enhanced production of reactive oxygen species (ROS), which are highly reactive oxygen-derived small molecules.^20–22^ While ROS are crucial in promoting essential cell functions, including the adipogenic commitment,^17^^,^^19^^,^^22^ levels are finely regulated by cellular detoxification systems such as antioxidant enzymes: superoxide dismutases (SOD), catalase (CAT), peroxiredoxins (PRDX), and glutathione peroxidases (GPX).^21^^,^^22^ Furthermore, growing evidence indicates that MSCs exert antioxidant properties that function buffering ROS within their local environment and facilitate intercellular communication regarding redox balance.^23^ This redox secretome of MSCs aims to mitigate oxidative stress.^6^^,^^7^

Redox changes occur in parallel to morphological and metabolic transformations during adipogenesis: during later maturation (day 6-21), they lose their fibroblastic morphology, accumulate triglycerides, and acquire mature adipocyte’s appearance and metabolic features.^24^ Triglyceride accumulation is closely associated with a coordinated rise in the expression of the enzymes involved in fatty acid biosynthesis, leading to an elevated rate of de novo lipogenesis and adipokine secretion.^25–28^ Under metabolic stress, mature adipocytes can engage inflammatory activation.^26^ Collectively, these tightly coordinated redox and metabolic adaptations suggest that maternal obesity may perturb the extracellular signaling landscape of fetal MSCs.

In our recent studies, we evaluated the redox state in MSCs and uncovered hallmarks of oxidative stress, coupled with higher adipogenic commitment in MSCs derived from neonates of mothers with obesity compared to those from mothers with normal-weight.^11^^,^^29^ Building upon these findings, we hypothesize that the impact of an obesogenic intrauterine environment on fetal MSCs metabolism might subsequently shape their paracrine redox and adipogenic activity within their surrounding environment. In this context, the present exploratory study aimed to explore extracellular proteomic signatures that account for the early adipogenic programming of MSCs in the offspring of women with obesity.

## Materials and methods

### Experimental design

MSCs derived from neonates born to normal-weight mothers (NW-MSCs) and from mothers with obesity (OB-MSCs) were subjected to *in vitro* adipogenesis. The secretome of these cells was assessed at various time points: on day 0 (MSCs, progenitor cells), day 5 (early adipogenic commitment, since we have recently evaluated early markers)^29^ and day 21 (upon reaching maturity as MSC-derived *in vitro* adipocytes, Figure S1). The analysis focused on characterizing the secretome during the three timepoints, after which we specifically focused on proteins associated with redox mechanisms and adipogenic profile at each time point.

### Subjects

Umbilical cords were obtained from the placentas of women with obesity or normal weight, who delivered at the University Medical Centrum Groningen (UMCG) maternity ward, Groningen, The Netherlands, from July 2021 to February 2023. Umbilical cords were considered as biological waste material following routine childbirth procedures and no personal donor information was collected. The study complied with all relevant institutional and national ethical regulations regarding the use of biological materials. A pregestational maternal body mass index (BMI) >30 kg/m^2^ was considered for the group of women with obesity (OB, n = 3) and 18.5-24.5 kg/m^2^ for women with normal weight (NW, n = 3). The inclusion criteria included women >18 years, single and term pregnancies (>37 weeks). The exclusion criteria included women with gestational diabetes, preeclampsia, preterm birth, and neonatal complications. All participants remained anonymous, only the woman’s pregestational BMI (weight, height) and gestational weight were recorded.

### Isolation of Wharton’s jelly-derived MSCs

Umbilical cords were obtained and immediately processed to get MSCs primary cultures. MSCs were isolated using the explant method.^11^^,^^16^ The umbilical cord was washed in cold Dulbecco’s Phosphate Buffered Saline (DPBS, Gibco, Thermo Fischer Scientific) and cut into 3 cm long pieces inside a laminar flow cabinet. Each piece was cut longitudinally, and blood vessels were discarded. Wharton’s jelly explants were plated and cultured with Dulbecco’s modified Eagle medium containing 10 mM glucose (DMEM, Gibco, Thermo Fischer Scientific), 10% Fetal calf serum (FCS, Sigma-Aldrich), 5.000 UI/mL Penicillin-Streptomycin (Gibco, Thermo Fischer Scientific), and maintained at 37°C in 5% CO_2_. Media were changed every four days, and culture was maintained for 10-14 days. By this time, a solid population of cells had sprouted out from the explants and had covered the explant perimeter. Subsequently, the sprouted cells were trypsinized (Gibco, Thermo Fischer Scientific) and expanded into further passages by seeding at 4000/cm^2^ in culture flasks. The medium was changed every three days, and cells were maintained in the same culturing conditions. Cells were passaged after six days of culture reaching 80% confluency (passages 1-2) and previously characterized for MSCs markers, including CD73, CD90, CD105+ and CD11b, CD34, and CD45−.^11^ Unless otherwise stated, all experiments were performed in cells from passage 2.

### Cell culture and adipogenic differentiation

Cells were seeded at 7000 cells/cm^2^ in 6-well plates and cultured with DMEM (Gibco, Thermo Fischer Scientific) (10 mM glucose), 10% FCS (Sigma-Aldrich), 5000 UI/ml Penicillin-Streptomycin (Gibco, Thermo Fischer Scientific) and maintained at 37°C in 5% CO_2_. At time point 0, cells were subjected to serum-free medium incubation for 24 hours. Subsequently, the supernatant was collected and centrifuged at 400×*g* for 10 minutes and stored at −80°C. In parallel, adipogenic differentiation was induced by culturing cells with high glucose DMEM + 10% FCS, 5000 UI/ml Penicillin-Streptomycin (Gibco, Thermo Fischer Scientific), insulin (1 nM), dexamethasone (0.1 μM) and 3-isobutyl-1-methylxantine (0.5 mM).^30^ Adipogenic differentiation was carried out for either 5 or 21 days. At each time point, cells were subjected to serum-free medium incubation for 24 hours to collect supernatant as described. Protein concentrations in cells were determined using the bicinchoninic acid (BCA) protein assay kit (Pierce, Thermo Fisher Scientific) (Table S1).

### Supernatant for secretome measurements and SP3 digestion

Protein concentrations in all supernatants were determined using the bicinchoninic acid (BCA) protein assay kit (Pierce, Thermo Fisher Scientific). The further processing of the supernatants followed a SP3 digestion method.^31^ Briefly, 25 μL of supernatant underwent reduction by adding dithiothreitol (10 mM) and were incubated for 30 min at 57°C. Subsequent alkylation of the sample was performed by adding iodoacetamide (30 mM) and incubating for 30 minutes at room temperature in the dark. Next, proteins were bound to prewashed Cytiva SeraMag beads (1:1 of hydrophilic and hydrophobic beads, Fisher Scientific) and diluted to 50% acetonitrile. After the removal of acetonitrile, the beads were washed with 80% (v/v) ethanol and acetonitrile. The beads were digested overnight with 40 μL of trypsin (2.5 ng/μL, Promega) at 37 °C. Lastly, 10 μL of 1% (v/v) formic acid were added to stop the digestion, and untargeted proteomics analyses of the sample were further processed on the Evosep (Evosep Biosystems).^31^

***Liquid chromatography–mass spectrometry analysis.*** Mass spectrometry analyses were performed with a quadrupole orbitrap mass spectrometer (Orbitrap Exploris 480, Thermo Scientific). Peptide separation was achieved through liquid chromatography (Evosep One, Evosep), with a nano-LC column (EV1137 Performance column, Evosep). 10% of the sucrose fraction digests were subjected to separation utilizing the 30SPD workflow (Evosep). The mass spectrometer was configured in positive ion mode and operated in data-independent acquisition (DIA) mode. Isolation windows of 16 *m*/*z* were employed with a precursor mass range of 400-1000. Additionally, FAIMS alternated between CV −45 and −60 V, incorporating three scheduled MS1 scans during each precursor mass range screening.^31^

### Data analyses

Raw liquid chromatography-mass spectrometry (LC-MS) data were processed with Spectronaut (18.3.230830) (Biognosys), with the standard settings of the direct DIA workflow. MS1-level quantification was conducted using the human SwissProt database (www.uniprot.org, 20 422 entries). We employed local normalization for quantification of the data, and Q-value filtering was performed using the traditional setting without imputation. Proteins not detected on any specific day were excluded from further analysis. A protein was considered present if it was detected in at least two-thirds of the samples within a given group and day. Analysis of enrichment pathways was performed by Metascape considering a *P*-value cutoff of .01 and an enrichment factor of 1.5.^32^ For general characterization of the secretomes, we considered only common proteins between NW-MSCs and OB-MSCs. Further, data was transformed to log2 and Mann-Whitney *U* tests were applied for each protein found in the secretome of NW-MSCs and OB-MSCs, to obtain differentially regulated proteins (upregulated or downregulated proteins, cut-off log2 ratio = 0.5). For downstream analysis, we considered only the differentially regulated proteins (candidate proteins). A detailed overview of the proteomic data is provided in Table S2, and the analysis workflow is provided in Figure S2.

### Enrichment pathway analyses

Candidate proteins were assessed for enrichment pathway analyses with Metascape,^32^ considering parental Gene Ontology pathways for comparisons of OB-MSCs versus NW-MSCs on day 0, 5 and 21 (*P*-value cutoff = .01; adjusted with adjusted with Benjamini-Hochberg correction to control the false discovery rate, FDR). Analyses included Gene Ontology, Reactome, and KEGG databases.

### Redox proteins

The list of candidate proteins from days 0, 5, and 21 was evaluated in STRING databases^33^ considering GO Biological Process: response to oxidative stress (GO: 0006979; FDR 0.00091), regulation of cellular response to oxidative stress (GO: 19000407; FDR 0.0075), response to reactive oxygen species (GO: 000302; FDR 0.0096), cell redox homeostasis (GO: 0045454, FDR 0.0168), glutathione metabolic process (GO: 0006749; FDR 4.55e−06) and NAD metabolism (GO: 0019674; FDR 0.0343).

### Adipogenic proteins

The list of candidate proteins for days 0, 5, and 21 was evaluated in STRING databases for GO Biological Process: Cellular lipid metabolic process (GO: 0044255, FDR 0.0238), Triglyceride homeostasis (GO: 0070328, FDR 0.0025), Lipid storage (GO: 0019915; FDR 0.0049), Lipid homeostasis (GO: 0055088, FDR 0.0016). Reactome/KEGG pathways: Transcriptional regulation of white adipocyte differentiation (HAS-381340; FDR 0.0329), PPAR signaling pathway (hsa033320; FDR 0.0060), Regulation of Insulin-like Growth Factor (IGF) transport proteins (HSA-381426; FDR 1.36e−20), WikiPathways: Adipogenesis (WP236; FDR 0.00265). For adipokines, proteins were identified according to reported adipose-derived secreted factors with endocrine function.^26–28^

### Statistical analysis

For secretomic analysis, volcano plots were generated using non-parametric Mann-Whitney *U* tests (to highlight trends in protein abundance between groups). To control for multiple comparisons across the full secretome, p-values were adjusted using a permutation-based false discovery rate (FDR). Volcano plots were generated using the ggplot2 package, and heat maps were generated using the gplots package in R Studio version 3.5.1. For heat maps, data was transformed to log2 and converted to z-score, and hierarchical clustering was applied to proteins and samples. For targeted analyses of antioxidant enzymes, lipid localization, fatty acid synthesis proteins, and adipokines, data were expressed as median values and 25th and 75th percentiles in Graphpad Prism (GraphPad Inc.). Normality was assessed with the Shapiro-Wilks test. Data was log2 transformed and a Two-way analysis of variance (ANOVA) followed by Tukey’s post hoc test was used to explore trends across independent variables including BMI (NW-MSCs versus OB-MSCs) and days of differentiation (0, 5, 21). *P* values < .05 were considered indicative of trends in the analysis.

## Results

### General characterization of the secretome of NW-MSCs and OB-MSCs

**Figure 1.**
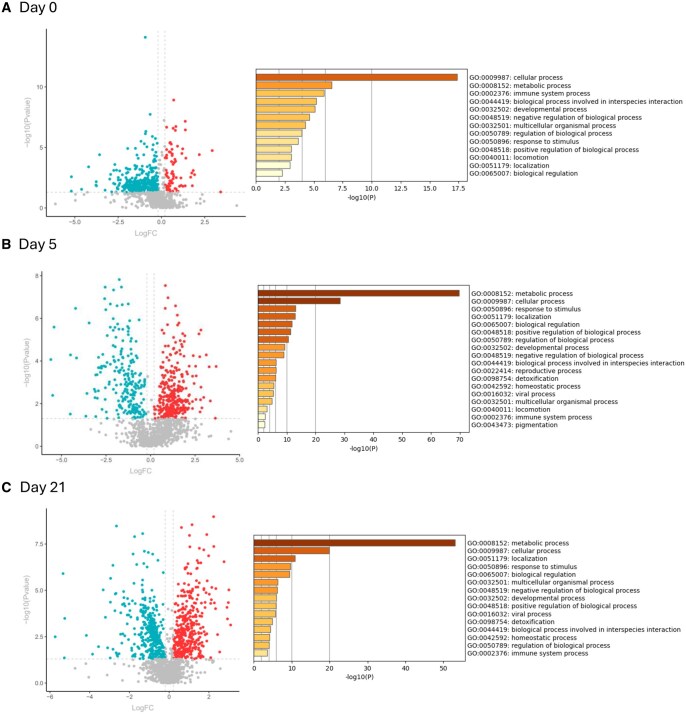
Characterization of the secretome from OB-MSCs versus NW-MSCs on days 0, 5, and 21 of adipogenesis. (A-C) Volcano plots showing differentially secreted proteins in the secretome of OB-MSCs relative to NW-MSCs. Log2 fold changes (LogFC, *P* value = .05) are represented for downregulation (blue), upregulation (red) and non-changed (gray) common proteins for days 0, 5, and 21 of adipogenesis. Top enrichment pathways identified by Metascape upon analysis of the upregulated and downregulated proteins in the secretome of OB-MSCs relative to NW-MSCs for each timepoint. Analyses were performed considering Gene Ontology Biological Processes terms (*P*-value cutoff = 0.01, enrichment factor of 1.5).

We analyzed the proteins of the secretome of NW-MSCs and OB-MSCs. We found a total of 981 proteins on day 0, from which 96 were upregulated in OB-MSCs versus NW-MSCs (red, Figure 1A), 359 proteins were downregulated in OB-MSCs versus NW-MSCs (blue, Figure 1A), and 534 proteins that did not differ between groups (grey, Figure 1A). From the upregulated and downregulated proteins, GO Biological Process analysis returned the top three terms: cellular process (GO: 0009987), metabolic process (GO: 0008152) and immune system process (GO: 0002376) (red and blue, Figure 1A). For subclass analyses, the top 20 terms are detailed in Table S3. These findings suggest that OB-MSCs display altered regulation of immune-related and metabolic pathways compared with NW-MSCs, potentially reflecting an obesity-associated secretory phenotype.

We found 1215 proteins on day 5 in the secretome of NW-MSCs and OB-MSCs. From these proteins, 408 were upregulated (red, Figure 1B), 251 were downregulated (blue, Figure 1B), and 556 proteins did not change (grey, Figure 1B). When we evaluated the pathways represented by these upregulated and downregulated proteins, the top three GO Biological Process terms found were: metabolic process (GO: 0008152), cellular process (GO: 0009987), and response to stimulus (GO: 0050896) (red and blue, Figure 1B). For subclass analyses, the top 20 terms are detailed in Table S4. These results indicate that during early adipogenic differentiation, OB-MSCs secrete proteins more strongly associated with metabolic activity and stress responses compared to NW-MSCs.

Finally, our analyses evidenced 1506 proteins on day 21 of adipogenesis in the secretome of NW-MSCs and OB-MSCs. 331 proteins were upregulated (red, Figure 1C), 326 proteins were downregulated (blue, Figure 1C), and 849 proteins did not change (grey, Figure 1C). The top three GO Biological Process terms associated with these proteins were: metabolic process (GO: 0008152), cellular process (GO: 0009987) and localization (GO: 0051179) (red and blue, Figure 1C). The top 20 terms found for subclass analyses are detailed in Table S5. These secretome differences between NW-MSCs and OB-MSCs are largely linked to metabolic regulation and protein localization, suggesting that obesity modifies adipocyte secretome and thus, adipose tissue.

### Redox pathways in the secretome of NW-MSCs and OB-MSCs during adipogenesis

In this study we were interested in investigating the redox profile from the proteins in the secretome of NW-MSCs and OB-MSCs on days 0, 5, and 21 of adipogenesis. The GO Biological Process pathways that show an enriched expression on day 0, 5, and 21, were response to stimulus (GO: 0050896) and detoxification (GO: 0098754). This suggests a different redox secretome between both groups (Figure 1A-C). Subclass enrichment pathways are described in Table 1.

**Table 1.** Enrichment pathways of redox proteins in the secretome of OB-MSCs versus NW-MSCs on day 0, 5, and 21 of adipogenesis.

GO	Category	Description	Count	%	Log10(P)	Log10(q)
**Day 0**						
**GO: 0006979**	GO Biological Processes	Response to oxidative stress	15	62.50	−22.82	−18.57
**R-HSA-9711123**	Reactome Gene Sets	Cellular response to chemical stress	11	45.83	−17.39	−13.84
**GO: 0042743**	GO Biological Processes	Hydrogen peroxide metabolic process	7	29.17	−14.78	−11.54
**GO: 0006749**	GO Biological Processes	Glutathione metabolic process	5	20.83	−9.13	−6.17
**GO: 1902175**	GO Biological Processes	Regulation of oxidative stress-induced intrinsic apoptotic signaling pathway	4	16.67	−7.28	−4.43
**Day 5**						
**GO: 0006979**	GO Biological Processes	Response to oxidative stress	21	75.00	−34.48	−30.23
**GO: 1990748**	GO Biological Processes	Cellular detoxification	13	46.43	−24.43	−20.48
**GO: 0006749**	GO Biological Processes	Glutathione metabolic process	11	39.29	−23.20	−19.59
**GO: 0034599**	GO Biological Processes	cellular response to oxidative stress	11	39.29	−16.31	−13.01
**R-HSA-9711123**	Reactome Gene Sets	Cellular response to chemical stress	9	32.14	−12.62	−9.54
**Day 21**						
**GO: 0006979**	GO Biological Processes	Response to oxidative stress	12	85.71	−21.14	−16.89
**GO: 0034599**	GO Biological Processes	Cellular response to oxidative stress	8	57.14	−13.67	−9.72
**GO: 0098869**	GO Biological Processes	Cellular oxidant detoxification	5	35.71	−9.37	−5.89
**GO: 1902175**	GO Biological Processes	Regulation of oxidative stress-induced intrinsic apoptotic signaling pathway	3	21.43	−5.90	−2.90
**GO: 0043467**	GO Biological Processes	Regulation of generation of precursor metabolites and energy	3	21.43	−4.47	−1.63

**Figure 2.**
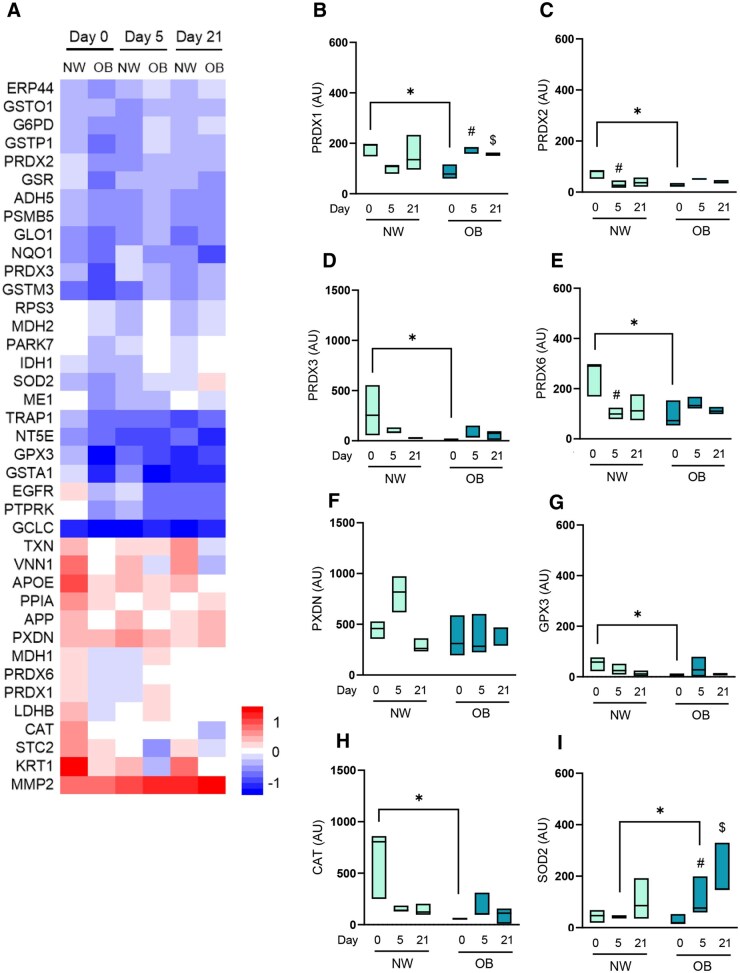
Redox proteins in the secretome of NW-MSCs and OB-MSCs during adipogenesis. (A) Abundance of redox proteins in the secretome of NW-MSCs and OB-MSCs for day 0, 5, and 21 of adipogenesis (relative z-score for log2 values). (B-I) Secreted antioxidant enzymes during day 0, 5, and 21 of adipogenesis in NW-MSCs and OB-MSCs. PRDX 1/2/3/6, peroxiredoxins; PXDN, peroxidasin; GPX3, glutathione peroxidase 3; CAT, catalase; SOD2, superoxide dismutase 2. Effect of obesity over the expression of PRDX3 and CAT (*P* = .04 and .007, respectively). Two-way ANOVA: Effect of day of differentiation over the expression of SOD2 (*P* = .006). Tukey’s range post testing: *P < .05 (NW-MSCs versus OB-MSCs); ^#^*P* < .05 (day 5 versus day 0). ^$^*P* < .05 (day 21 versus day 0).

We evaluated the proteins particularly associated to these redox pathways. The overall analysis for redox proteins on day 0 show that 23 proteins have decreased levels in the secretome of OB-MSCs compared to NW-MSCs. These decreased proteins include antioxidant enzymes such as PRDX1/3/6, CAT, as well as components of the glutathione system such as glutathione reductase (GSR) and glutathione S-transferase alpha 1 (GSTA1) (Figure 2A, Table S6). Interestingly, we found that during day 5 of adipogenesis, 18 redox proteins were increased in the secretome of OB-MSCs compared to NW-MSCs. These proteins included antioxidant enzymes, such as superoxide dismutase 2 (SOD2), PRDX1/2/6, and components of the glutathione system such as glutathione S-transferase P1 (GSTP1) and glutathione S-transferase O1 (GSTO1), among others redox mediators. Conversely, only four proteins were shown to be decreased in the secretome of OB-MSCs compared to NW-MSCs: peroxidasin homolog (PXDN), apolipoprotein E (APOE), stanniocalcin 2 (STC2) and keratin 1 (KRT1) (Figure 2A, Table S6). Further, on day 21 of adipogenesis, we identified seven redox proteins that were more abundant in the secretome of OB-MSCs compared to NW-MSCs, including PRDX6 and PXDN. We found four redox proteins decreased in the secretome of OB-MSCs compared to NW-MSCs: protein receptor-type tyrosine-protein phosphatase kappa (PTPRK), pantetheinase (VNN1), NAD(P)H dehydrogenase quinone 1 (NQO1) and KRT1 (Figure 2A, Table S6).

### Antioxidant enzymes in the secretome of NW-MSCs and OB-MSCs during adipogenesis

We analyzed the presence of antioxidant enzymes throughout adipogenesis (Figure 2B-I). Our analyses showed an effect of obesity on expression levels of PRDX3 and CAT (*P* = .04 and .007, respectively). The main antioxidant enzymes that were less abundant in the secretome of OB-MSCs compared to NW-MSCs were PRDX1/2/3/6, GPX3 and CAT (*P* < .05, Figure 2B-E, G-H). Interestingly, SOD2 secretion was increased the secretome of OB-MSCs compared to NW-MSCs (*P* < .05, Figure 2I). Further, there was an effect of day of differentiation over the expression levels of SOD2 (*P* = .006, Figure 2I).

### Adipogenic pathways in the secretome of NW-MSCs and OB-MSCs during adipogenesis

We found that cellular lipid catabolic process (GO: 0044242) and lipid localization (GO: 0010876) were the most enriched subclass pathways represented on days 0, 5, and 21 (Table 2).

**Table 2.** Enrichment pathways of adipogenic proteins in the secretome of OB-MSCs versus NW-MSCs on day 0, 5, and 21 of adipogenesis.

GO	Category	Description	Count	%	Log10(P)	Log10(q)
**Day 0**						
**GO: 0044242**	GO Biological Processes	Cellular lipid catabolic process	12	33.33	−17.35	−13.10
**GO: 1901615**	GO Biological Processes	Organic hydroxy compound metabolic process	14	38.89	−15.82	−11.86
**GO: 0019752**	GO Biological Processes	Carboxylic acid metabolic process	15	41.67	−14.29	−10.74
**GO: 0055088**	GO Biological Processes	Lipid homeostasis	10	27.78	−14.12	−10.64
**R-HSA-381426**	Reactome Gene Sets	Regulation of Insulin-like Growth Factor (IGF) transport and uptake by Insulin-like Growth Factor Binding Proteins (IGFBPs)	8	22.22	−11.73	−8.82
**Day 5**						
**GO: 0010876**	GO Biological Processes	Lipid localization	15	40.54	−18.73	−14.47
**GO: 0008202**	GO Biological Processes	Steroid metabolic process	13	35.14	−17.56	−13.61
**GO: 0019216**	GO Biological Processes	Regulation of lipid metabolic process	11	29.73	−12.90	−9.83
**R-HSA-556833**	Reactome Gene Sets	Metabolism of lipids	13	35.14	−11.56	−8.63
**GO: 0019752**	GO Biological Processes	Carboxylic acid metabolic process	12	32.43	−10.04	−7.24
**Day 21**						
**GO: 0010876**	GO Biological Processes	Lipid localization	18	52.94	−25.08	−20.82
**GO: 0019752**	GO Biological Processes	Carboxylic acid metabolic process	16	47.06	−16.26	−12.48
**GO: 0006066**	GO Biological Processes	Alcohol metabolic process	12	35.29	−15.06	−11.50
**GO: 0009725**	GO Biological Processes	Response to hormone	12	35.29	−10.56	−7.91
**GO: 1905952**	GO Biological Processes	Regulation of lipid localization	8	23.53	−10.56	−7.91

**Figure 3.**
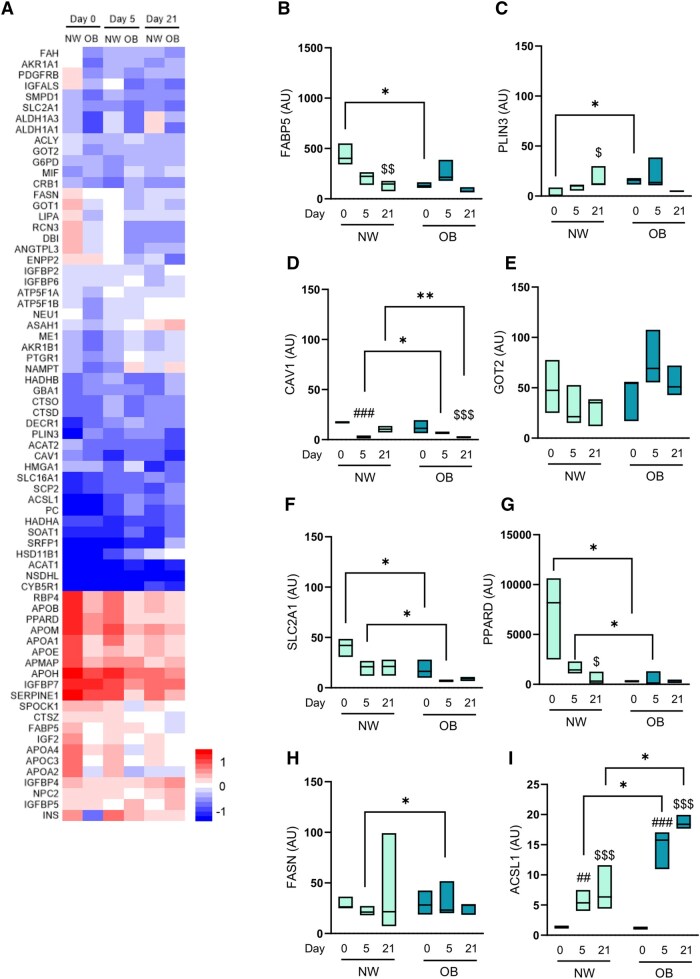
Adipogenic proteins in the secretome of NW-MSCs and OB-MSCs during adipogenesis. (A) Abundance of adipogenic proteins in the secretome of NW-MSCs and OB-MSCs for day 0, 5, and 21 of adipogenesis (relative z-score for log2 values). (B-G) Lipid localization proteins. (B) FABP5, fatty acid binding protein 5; (C) PLIN3, perilipin 3; (D) CAV1, caveolin 1, ANGPTL3, angiopoietin like protein 3; (E) GOT2, Glutamic-oxaloacetic transaminase; (F) SLC2A1, soluble carrier family 2 member 1; (G) PPARD, peroxisome proliferator-activator receptor delta. Two-way ANOVA: Effect of obesity on FABP5, SLC2A1 and PPARD (*P* = .01, .0004, and .0006, respectively). Effect of day of differentiation on FABP5, CAV1 and SLC2A1 (*P* = .001, .0001, and .04, respectively). (H-I) Fatty acid synthesis proteins. FASN, fatty acid synthase; ACSL1, acyl-COA synthase long chain 1. Two-way ANOVA: Effect of obesity on ACSL1 (*P* = .003). Effect of day of differentiation on ACSL1 (*P* = .0001). Tukey’s range post testing: *P < .05 (NW-MSCs versus OB-MSCs); **P < .01 (NW-MSCs versus OB-MSCs); ^##^*P* < .01 (day 5 versus day 0); ^###^*P* < .001 (day 5 versus day 0); ^$^*P* < .05 (day 21 versus day 0); ^$$^*P* < .01 (day 21 versus day 0); ^$$$^*P* < .001 (day 21 versus day 0).

In our analysis we identified four adipogenic proteins that were increased in the secretome of OB-MSCs compared to NW-MSCs on day 0: lysosomal acid glucosylceramidase (GBA1), macrophage migration inhibitory factor (MIF), trifunctional enzyme subunit beta (HADHB) and insulin-like growth factor binding protein 6 (IGFBP6). Conversely, 32 proteins were decreased in the secretome of OB-MSCs compared to NW-MSCs, including proteins involved in lipid metabolism (APOE; aldo-keto reductase 1 A1, AKR1A1; and prostaglandin reductase 1, PTGR1) and regulators of insulin pathway (insulin-like growth factor binding protein acid labile subunit, IGFALS; insulin-like growth factor-binding protein 2, IGFBP2), among others (Figure 3A, Table S7).

During adipogenesis, we identified 18 proteins with higher abundance in the secretome of OB-MSCs compared to NW-MSCs on day 5. These proteins were mainly related to lipid metabolism (visfatin/nicotinamide phosphoribosyltransferase, NAMPT; glucose-6-phosphate 1-dehydrogenase, G6PD), adipogenesis (high mobility group protein A1, HMGA1; and MIF), among other mechanisms. In contrast, 17 proteins were found with lower abundance in the secretome of OB-MSCs compared to NW-MSCs: proteins related to lipid metabolism (apolipoprotein A1, APOA2; and apolipoprotein A1, APOA1), regulation of insulin pathway (insulin, INS; and insulin-like growth factor binding protein 6 and 7, IGFBP6/7), and adipogenesis (testican 1, SPOCK1; and solute carrier 2 facilitated glucose transporter 1/GLUT1, SLC2A1) (Figure 3A, Table S7).

On day 21, our analyses revealed 16 proteins that were increased in the secretome of OB-MSCs compared to NW-MSCs, which were mainly related to lipid metabolism (sterol O-acyltransferase 1, SOAT1; and HADHB). Conversely, 18 proteins were decreased in the secretome of OB-MSCs compared to NW-MSCs, also related to lipid metabolism (aldehyde dehydrogenase 1 A1, ADH1A1; and caveolin-1, CAV1) (Figure 3A, Table S7).

### Lipid localization-related proteins in the secretome of NW-MSCs and OB-MSCs during adipogenesis

As indicated above, lipid localization (GO: 0010876) was identified as the most enriched subclass pathway from the adipogenic proteins in the secretome of OB-MSCs and NW-MSCs (day 5 and 21, Table 2). Consequently, we evaluated the temporal pattern of these proteins during adipogenesis. Our analysis showed an effect of obesity over the secretion of FABP5, SLC2A1, and PPARD (*P* = .01, .0004, and .0006, respectively). FABP5 was lower (*P* < .05, Figure 3B), while PLIN3 levels were higher in the secretome of OB-MSCs versus NW-MSCs (*P* < .05, Figure 3C). CAV1 levels fluctuated in the secretome of OB-MSCs compared to NW- (*P* < .05 and <.001 for days 5 and 21, respectively, Figure 3D). SLC2A1 and PPARD levels were decreased in OB-MSCs compared to NW-MSCs (*P* < 0.05, Figure 3F, G).

We found an effect of day of differentiation on the expression of fatty acid binding protein 5 (FABP5), CAV1, and SLC2A1 (*P* = .001, .0001, and .04, respectively, Figure 3B, D, F).

### Fatty acid synthesis-related proteins in the secretome of NW-MSCs and OB-MSCs during adipogenesis

Our analyses showed an effect of obesity over levels of ACSL1 (*P* = .003, Figure 3I). Further, our results showed that fatty acid synthase (FASN) levels were higher in the secretome of OB-MSCs compared to NW-MSCs (*P* < .05, Figure 3H). There was a higher abundance of ACSL1 in the secretome of OB-MSCs compared to NW-MSCs (*P* < .05, Figure 3I). Our subsequent analyses showed an effect of day of differentiation (*P* = .0001) over levels of long-chain-fatty-acid-CoA ligase 1 (ACSL1), but no effect over fatty acid synthase (FASN) levels (*P* = .08, Figure 3H-I).

### Adipokines in the secretome of NW-MSCs and OB-MSCs during adipogenesis

**Figure 4.**
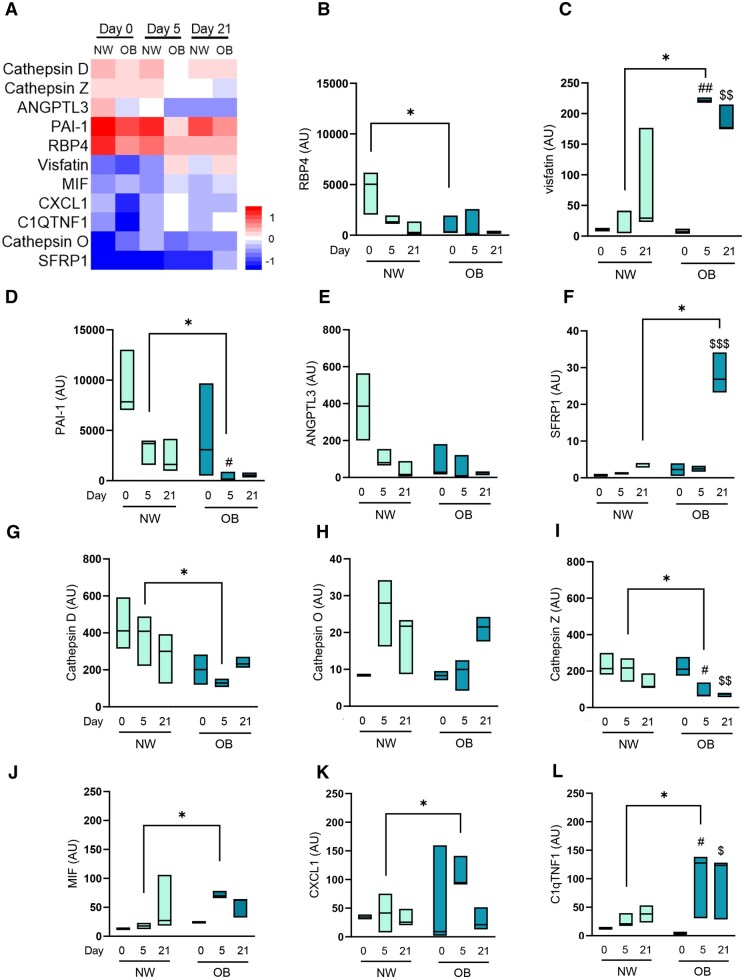
Adipokines found in the secretome of NW-MSCs and OB-MSCs during adipogenesis. (A) Abundance of secreted adipokines in the secretome of NW-MSCs and OB-MSCs for day 0, 5, and 21 of adipogenesis (relative z-score for log2 values). (B-L) Secreted adipokines in NW-MSCs and OB-MSCs for day 0, 5, and 21 of adipogenesis. RBP4, retinol-binding protein 4; ANGPTL3, angiopoietin-like protein 3; SFRP1, secreted frizzle related protein 1; MIF, macrophage inhibitory factor; PAI-1, plasminogen activator inhibitor 1; CXCL1, chemokine ligand 1; C1QTNF1, C1q tumor necrosis factor related protein 1. Two-way ANOVA: Effect of obesity over the expression of RBP4, visfatin/NAMPT, PAI-1, ANGPTL3, SFRP1, Cathepsin D, Cathepsin Z and MIF (*P* = .03, .01, .001, .003, .03, .004, .003, and .01, respectively). Effect of day of differentiation over levels of visfatin/NAMPT, PAI-1, ANGPTL3, SFRP1 and Cathepsin Z (*P* = .0007, .009, .004, .0015, and .0015, respectively). Tukey’s post testing: *P < .05 (NW-MSCs versus OB-MSCs); ^#^*P* < .05 (day 5 versus day 0); ^##^*P* < .01 (day 5 versus day 0); ^$^*P* < .05 (day 21 versus day 0); ^$$^*P* < .01 (day 21 versus day 0); ^$$$^*P* < .001 (day 21 versus day 0).

We identified the presence of retinol-binding protein 4 (RBP4), visfatin/NAMPT, plasminogen activator inhibitor 1 (PAI-1), and the chemokines C-X-C motif chemokine 1 (CXCL1) and complement C1q tumor necrosis factor-related protein 1 (C1QTNF1) (Figure 4A). We also found novel adipokines, including cathepsins, angiopoietin-related protein 3 (ANGPTL3), secreted frizzled-related protein 1 (SFRP1), and MIF (Figure 4A). Our analyses showed an effect of obesity over the expression of RBP4, visfatin/NAMPT, PAI-1, ANGPTL3, SFRP1, Cathepsin D, Cathepsin Z and MIF (*P* = .03, .01, .001, .003, .03, .004, .003, and .01, respectively). We evidenced lower RBP4 levels in OB-MSCs versus NW-MSCs secretome (*P* < .05, Figure 4B). Interestingly, visfatin/NAMPT, MIF, CXCL1, and C1QTNF1 were further increased, while PAI-1, cathepsins D and Z were decreased in the secretome of OB-MSCs compared to NW-MSCs (*P* < .05, Figure 4C, 4J-L, *4D*). On day 21, SFRP1 levels were increased in the secretome of OB-MSCs compared to NW-MSCs (*P* < 0.05, Figure 4F). Notably, we found the presence of interleukin 6 (IL-6) in the secretome of OB-MSCs on day 0, while no expression was detected in NW-MSCs (data not shown).

Additionally, our results showed an effect of day of differentiation on expression levels of visfatin/NAMPT, PAI-1, ANGPTL3, SFRP1, and Cathepsin Z (*P* = .0007, .009, .004, .0015, and .0015, respectively, Figure 4C-F).

## Discussion

In this study, we characterized the secretome of NW-MSCs and OB-MSCs to gain preliminary insights into how maternal obesity could influence cellular status and cell signaling within the fetal adipose tissue. Our data reveals that MSCs are indeed modulated by maternal obesity, which influences their secretome. Thus, this could determine intercellular communication.^34–36^ Pathway analyses revealed fundamental differences between both groups, associated with a change in cellular and metabolic pathways on different stages of adipogenesis. Our results highlight the decreased expression of redox proteins on OB-MSCs as compared to NW-MSCs as progenitor cells (day 0), while showing increased expression of proteins associated with lipid synthesis and proinflammatory adipokines, in early and late adipocytes (day 5 and 21), in OB-MSCs compared to NW-MSCs.

The enrichment of the pathway: response to oxidative stress (GO: 0006979), along with the reduced expression of redox proteins in the secretome of OB-MSCs compared to NW-MSCs such as peroxidins, CAT, and GPX3, suggests a decreased antioxidant potential of OB-MSCs within the extracellular space. Our findings are consistent with animal studies reporting decreased redox protein levels in the secretome of bone marrow-derived MSCs from high-fat diet mice. In particular, Ayaz-Guner et al. found an absence of glutamate-cysteine ligase (GCL), PRDX5, and PRDX6 compared to normal diet mice.^36^ Altogether, these results indicate that maternal obesity negatively impacts the extracellular redox balance of their offspring’s MSCs. Given that adipose tissue also contains undifferentiated MSCs,^37^ our data suggest that these cells may exhibit reduced detoxification capacity in neonates from mothers with obesity, potentially contributing to the oxidative stress-related pathophysiology of obesity.^11^^,^^38^

On the contrary, we observed that OB-MSCs show an increased secretion of redox proteins during day 5 and 21 of adipogenic differentiation, compared to NW-MSCs. These results can be related to the fact that mitochondrial metabolism and oxidative stress have been implicated in the differentiation of MSCs and adipogenic commitment,^18^ which is associated to an activation of antioxidant mechanisms.^39^ Among the redox proteins in the secretome between NW-MSCs and OB-MSCs, we found that Parkinson’s disease protein 7 (PARK7) and mitochondrial heat shock protein 75 kDa (TRAP1) were significantly decreased on day 0 but increased on day 5 and 21 of adipogenesis in OB-MSCs versus NW-MSCs. PARK7 is known to act as a redox sensor and can be secreted to promote protection against oxidative stress through intercellular ERK1/2 activation,^40^^,^^41^ while TRAP1 is a mitochondrial chaperone protein that has been reported to maintain mitochondrial homeostasis.^42^ While the intracellular functions of these proteins are well-characterized, it remains unclear to what extent their secretion might impact cellular and adipose tissue function. Nevertheless, our results suggest that PARK7 and TRAP1 could play an important role in regulating mitochondrial ROS levels during adipogenesis in the context of obesity. Notably, we have previously reported elevated mitochondrial ROS in OB-MSCs compared to NW-MSCs,^11^ and further studies should unveil the intracellular activity of PARK7, TRAP1 and ROS during adipogenesis.

In this study, we found that secreted SOD2 did not change during adipogenesis for NW-MSCs. However, it was increased during adipogenic induction in OB-MSCs. SOD2 is a mitochondrial antioxidant enzyme known to be upregulated during adipogenesis.^43^^,^^44^ Moreover, it has been described that under oxidative stress, cells are able to excrete mitochondrial cargo material through extracellular vesicles.^45^^,^^46^ Therefore, our findings suggest that OB-MSCs are under oxidative stress during adipogenic commitment, which could explain the higher secretion of SOD2 into the extracellular space.

We found lower peroxiredoxins in the secretome of OB-MSCs versus NW-MSCs on day 0 (PRDX1, PRDX2, PRDX3, and PRDX6), suggesting a lower antioxidant capacity. Peroxiredoxins are a family of cysteine-dependent peroxidase enzymes that regulate peroxide levels^47^ and their secretion has been described as a protective mechanism within the MSCs environment,^48^^,^^49^ therefore, OB-MSCs could have a less efficient antioxidant system compared to NW-MSCs. It is noteworthy to mention that PRDX2 and PRDX6 were decreased on day 5 compared to day 0 in the secretome of NW-MSCs, while this was not evidenced in OB-MSCs. This observation could be explained by the fact that MSCs are known to have important detoxification properties within their extracellular milieu,^23^^,^^47^^,^^48^ while mature adipocytes do not have this function. Therefore, this paracrine capacity could be reduced in healthy preadipocytes, and adipocytes (day 5 and 21) compared to healthy MSCs (day 0) in NW-MSCs.^48^ On the contrary, we observed a higher secretion of PRDX1 on day 5 compared to day 0 in OB-MSCs. This suggests that the lower levels of peroxiredoxins in OB-MSCs compared to NW-MSCs on day 0, could be compensated during early adipogenesis in OB-MSCs, thereby enhancing antioxidant capacity during adipogenic commitment.

Significant differences were found between NW-MSCs and OB-MSCs secretomes in the pathway: lipid localization (GO: 0010876). Particularly, PPARD was decreased in the secretome of OB-MSCs compared to NW-MSCs. PPARD is a nuclear receptor that plays an important role in lipid metabolism by promoting fatty acid oxidation,^49^ and the overexpression of PPARD has been associated with a decrease in body weight, adipocyte triglyceride accumulation, circulating free fatty acids and circulating triglyceride levels.^50^ Thus, our results suggest a possible increase in triglyceride accumulation in MSCs and preadipocytes, and future studies should evaluate the intracellular expression of PPARD in these cells. This is in line with the higher abundance of proteins related to fatty acid synthesis in the secretome of OB-MSCs: we found higher secretion of FASN and ACSL1 compared to NW-MSCs on days 5 and 21, indicating higher cellular lipid synthesis.^51^^,^^52^ In addition, this could be related to an altered lipid trafficking and droplet dynamics, as seen by altered levels of FABP5, PLIN3, CAV1, and SLC2A1 in OB-MSCs. It is also in line with higher adipogenic potential of OB-MSCs as shown by the higher lipid accumulation, PPARγ expression, bigger lipid droplet size and adipocyte hypertrophy.^11^^,^^29^^,^^53^ Collectively, these data suggest an altered lipid metabolism in OB-MSCs compared to NW-MSCs during adipogenesis and highlights potential targets in the secretome for further investigation into the mechanisms underlying metabolic dysfunction in the progeny.

In our study, we aimed to evaluate the secretion of adipokines during adipogenesis. Interestingly, our analysis did not detect classic adipokines such as leptin and adiponectin. While other studies show controversial results on their presence, mainly attributed to the experimental conditions and technical approach.^48^^,^^54–57^ This discrepancy highlights the importance of considering both the source of MSCs and the methodologies employed for adipogenic differentiation, when addressing these findings. Despite the absence of classical adipokines, we identified several novel adipokines: Notably, SFRP1 was more abundant in the secretome of OB-MSCs compared to NW-MSCs on day 21 of differentiation. SFRP1 is a proadipogenic peptide that inhibits the Wnt/β-catenin signaling pathway and is upregulated in the early stages of obesity.^58^ At day 5, considered an early stage of adipogenesis, higher levels of visfatin/NAMPT, MIF, CXCL1 and C1qTNF1 were detected in the secretome of OB-MSCs compared to NW-MSCs. These adipokines are associated with proinflammatory activity and have been linked to obesity and type II diabetes.^59–63^ It is important to note that higher levels of visfatin/NAMPT has been linked not only to inflammation but to metabolic dysfunction by activation of NF-*κ*B/STAT3 signaling pathways.^62^ In general, the dynamic regulation of these adipokines during adipogenesis indicate a potential proinflammatory state of adipose tissue and could affect metabolic regulation.^64^^,^^65^ This observation is particularly relevant, as it may reflect inflammatory processes occurring in fetal adipose tissue when exposed to maternal obesity. Our findings support the hypothesis that maternal obesity can promote a proinflammatory intrauterine environment, potentially imprinting adipose tissue inflammation. It is important to note that MSCs are present in neonatal adipose tissue and later in life, therefore, the proinflammatory secretome of OB-MSCs could contribute to metabolic dysfunction in postnatal life.

A key limitation of this study is the small sample size, which reduces statistical power and limits the ability to detect subtle differences in the secretome. As a result, the findings should be interpreted as exploratory and hypothesis-generating rather than confirmatory. These samples represent rare neonatal MSC from mothers with well-characterized body mass index profiles, making larger cohorts difficult to obtain. Despite this limitation, we observed consistent trends in key pathways, particularly those related to redox regulation, lipid metabolism, and proinflammatory signaling. These trends provide biologically meaningful insight into the potential impact of maternal obesity on neonatal MSC secretomes. Future studies with larger cohorts and complementary validation approaches will be necessary to confirm these observations and fully characterize their biological significance.

In conclusion, this study identified differences in the redox and adipogenic proteomic profiles in the secretome of OB-MSCs and NW-MSCs, highlighting early adipose tissue programming. Maternal obesity led to alterations in the secretome of progenitor cells at early stages of adipogenesis, favoring a proinflammatory shift to adipocyte development. Furthermore, we identified a downregulation of redox-associated pathways at the MSC stage, emphasizing the contribution of redox state to the dysfunctional adipose tissue microenvironment in the context of obesity. These findings support the concept that maternal obesity could imprint metabolic characteristics in offspring by modifying cellular environments, potentially influencing the future metabolic health of adipose tissue.

## Supplementary Material

sxag001_Supplementary_Data

## Data Availability

Data will be made available on request.
