# Supplementary figures and images for: Profiling the secretome: maternal obesity impacts redox and adipogenic signaling during neonatal mesenchymal stem cell adipogenesis
Source: Stem Cells. 2026 Jan 10;44(4):sxag001. doi: 10.1093/stmcls/sxag001 (PMC13026406; doi:10.1093/stmcls/sxag001)

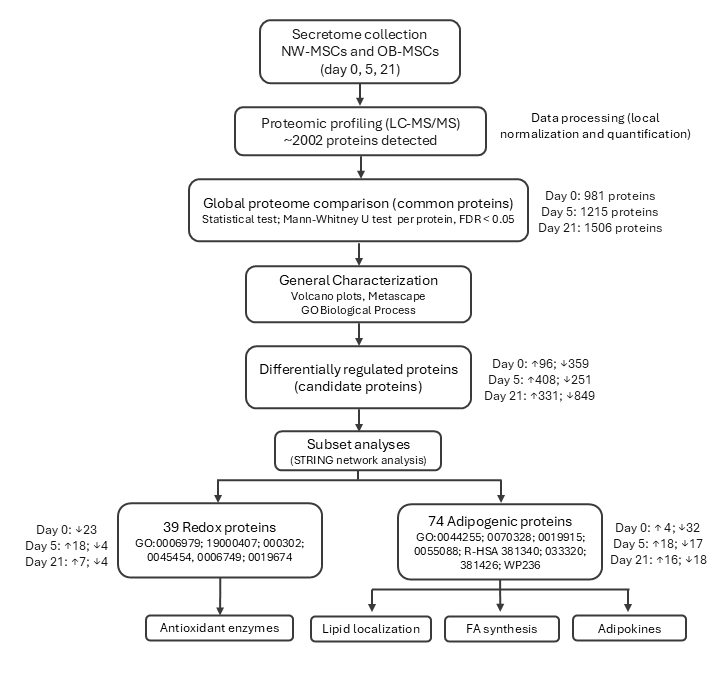

Supplement: sxag001_Supplementary_Data [file sxag001_supplementary_data.zip › Supl Figure S2.TIF]

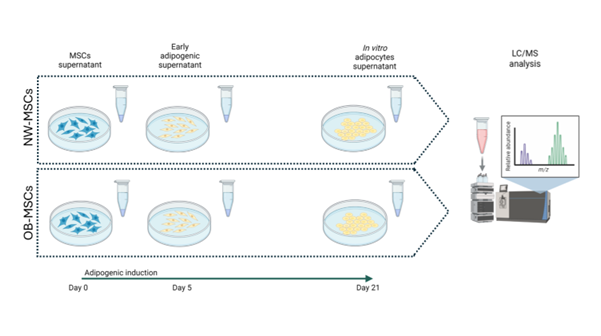

Supplement: sxag001_Supplementary_Data [file sxag001_supplementary_data.zip › Supl Figure S1.TIF]
